# Phosphorus and Iron Deficiencies Influences Rice Shoot Growth in an Oxygen Dependent Manner: Insight from Upland and Lowland Rice

**DOI:** 10.3390/ijms18030607

**Published:** 2017-03-10

**Authors:** Jenjira Mongon, Nanthana Chaiwong, Nadia Bouain, Chanakan Prom-u-thai, David Secco, Hatem Rouached

**Affiliations:** 1 Biochimie et Physiologie Moléculaire des Plantes Research Unit, Institut National de la Recherche Agronomique—Centre National de la Recherche Scientifique—Montpellier University, 34060 Montpellier, France; jenjira@g.swu.ac.th (J.M.); nantana.c189@gmail.com (N.C.); nadia.bouain@gmail.com (N.B.); david.secco@gmail.com (D.S.); 2 Bodhivijjalaya College, Srinakharinwirot University, 10110 Bangkok, Thailand; 3 Agronomy Division, Department of Plant and Soil Sciences, Faculty of Agriculture, Chiang Mai University, 50200 Chiang Mai, Thailand; chanakan15@hotmail.com

**Keywords:** rice, phosphate, iron, oxygen, signaling crosstalk

## Abstract

Rice is the main staple crop for one-third of the world population. To maximize yields, large quantities and constant input of fertilizers containing essential nutrients such as phosphorus (P) and iron (Fe) are added. Rice can germinate in both aerobic and anaerobic conditions, but the crosstalk between oxygen (O_2_) and nutrients such as P and Fe on plant growth remains obscure. The aim of this work was to test whether such interactions exist, and, if so, if they are conserved between up- and lowland rice varieties. To do so, we assessed shoot and root biomass as well as inorganic phosphate (Pi) accumulation in four rice varieties, including two lowland rice varieties Nipponbare and Suphanburi 1 (SPR1) (adapted to non-aerated condition) and two upland rice varieties CMU122 and Sew Mae Jun (SMJ) (adapted to aerated condition) under various conditions of Pi and/or Fe deficiencies, in aerated and non-areated solution. Under these different experimental conditions, our results revealed that the altered shoot biomass in Nipponbare and SPR1 was O_2_-dependent but to a lesser extent in CMU122 and SMJ cultivars. In this perspective, discovering the biological significance and molecular basis of these mineral elements and O_2_ signal interaction is needed to fully appreciate the performance of plants to multiple environmental changes.

## 1. Introduction

Rice (*Oryza sativa* L.) is one of the most important staple foods worldwide. Depending on the water regime cultivation system, rice can be divided into lowland rice and upland rice. Lowland rice is grown in flood-prone areas and predominates in Asia where it represents 70% of the total rice production [1]. In these flooded soils, the water layer limits O_2_ diffusion contributing to the creation of hypoxic growth conditions [2,3]. In opposition, upland rice is grown in rainfed areas where soils are usually well drained [4]. The effect of low O_2_ on rice growth is fairly well documented at the root level and, to a much less extent, on shoots [3,5,6,7]. Briefly, lowland rice evolved different strategies to cope with O_2_ deficiency such as the development of aerenchymas within the roots to transport O_2_ from shoot base to root tips [3,8,9]. Some of the upland rice varieties (IAC1131 and IR3880-5) had less root porosity (% gas spaces per unit tissue volume) than lowland rice when grown in a stagnant deoxygenated solution [10], which may account for the lower efficiency of upland rice to cope with low O_2_ condition. However, in contrast to root tissues, the effects of O_2_ availability in the soil on rice shoot biomass production in upland rice and lowland rice remain poorly investigated.

The growth and development of rice depends also on the availability of essential macro- and micronutrients such as Phosphorus (P) and iron (Fe), respectively. P is a component of vital biological molecules including nucleic acids, energy sources, phospholipids, and phytic acid [11], and is also involved in the photosynthetic activity [11]. As a result, the content of P is in the range of 0.43%–0.83% of rice dry matter [12], and P starvation results in plants with stunted growth. Fe, an essential micronutrient for cell functioning, plays a cofactor role in metabolic pathways, especially in the photosynthetic activity [13]. It is thus not surprising to observe that P or Fe deficiency alters plant growth. Intriguingly, recent research showed that P and Fe homeostasis could interact in an antagonistic manner to modulate growth of rice shoots [14]. For instance, Fe deficiency severely affects shoot growth of rice (lowland), which can be reverted by simultaneous Fe and P deficiency [14]. Fe deficiency also increases the phosphate (Pi) concentration in shoots [14].

In its natural habit, rice can simultaneously encounter nutrient (P and Fe) and O_2_ stresses. In this context, this study was designed to assess the existence and possible influence of the interaction between O_2_, Pi and Fe availability on shoots and roots biomass in four contrasting rice genotypes: upland rice (Nipponbare and Suphanburi 1 (SRP1)) and lowland rice (CMU122 and Sew Mae Jun (SMJ)). We also determined the Pi content in roots and shoots of each of these genotypes in all tested conditions. Our phenotypic analysis revealed an interesting interplay between the three elements Pi–Fe–O_2_ to influence the shoot growth of the four rice varieties in a contrasting manner. These results pave the way towards further genetic research works to uncover the molecular basis of Pi-Fe-O_2_ in rice varieties.

## 2. Results

In order to identify the effects of single and combined mineral nutrient deficiencies in aerated and non-aerated solutions, four different rice genotypes were used: two lowland rice varieties (Nipponbare and SRP1) and two upland rice varieties (CMU122 and SMJ). To do so, all genotypes were grown in full nutrient conditions, −Pi, −Fe and −Pi-Fe in hydroponics for 10 days with or without bubbling the solution (aerated and non-aerated), and then biomass as well Pi concentration were assessed in shoots and roots.

**Figure 1:**
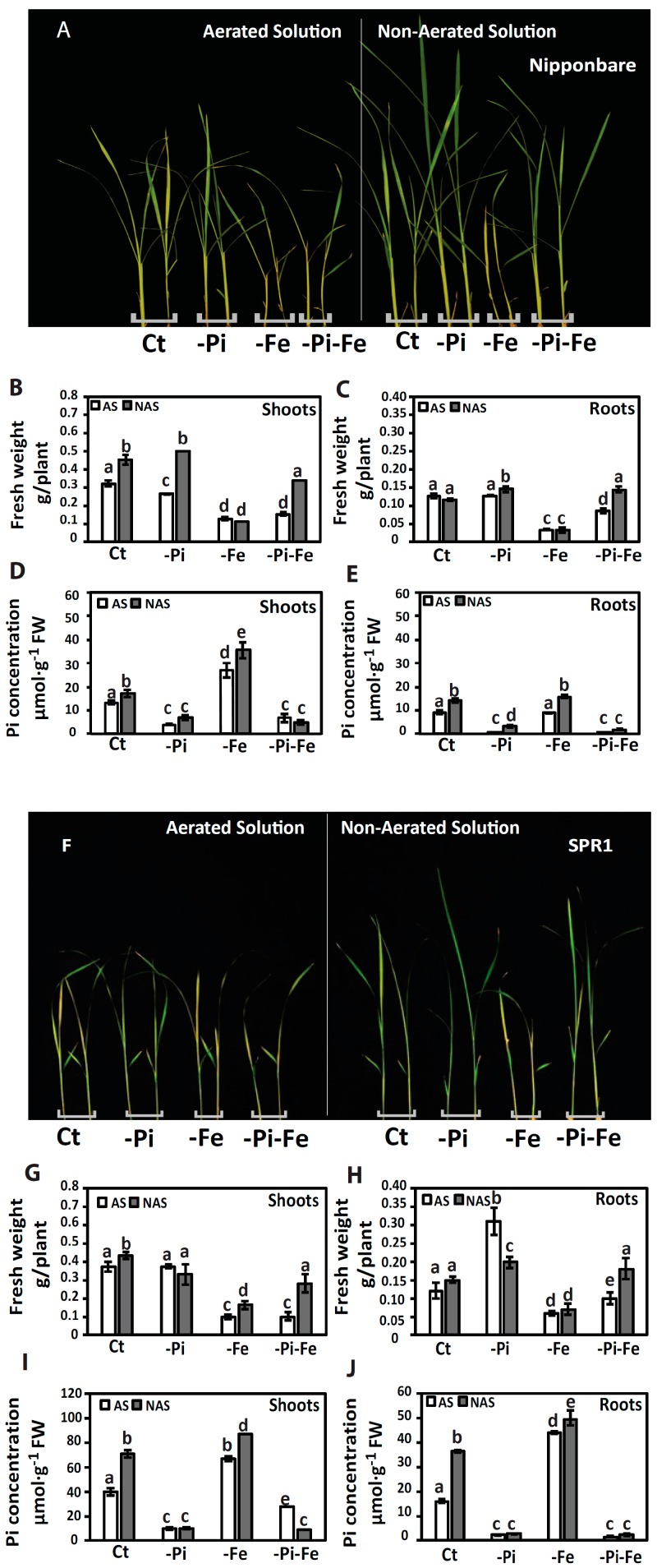
Responses of lowland rice (Nipponbare, Suphanburi 1 (SRP1) varieties) seedlings grown under various O_2_ and nutrient deficiency conditions. Ten-day-old seedlings were transferred to one of the combinatory treatments consisting of O_2_ (aerated solution (AS) and non-aerated solution (NAS)) nutrient treatments (full strength nutrient solution (Ct), phosphate deficiency (−Pi), iron deficiency (−Fe) and Pi and Fe deficiency (−Pi-Fe)). (**A**,**F**) phenotypes of seedlings after 10 days of treatment; (**B**,**G**) shoots and (**C**,**H**) roots biomass of seedlings after 10 days of treatment; (**D**,**I**) shoots and (**E**,**J**) roots Pi concentration of seedlings after 10 days of treatment. The data are given as mean ± s.e. (*n* = 3). Letters a, b, c, d, e on histograms indicate statistical significance at *p* < 0.05. FW, fresh weight.

**Figure 2:**
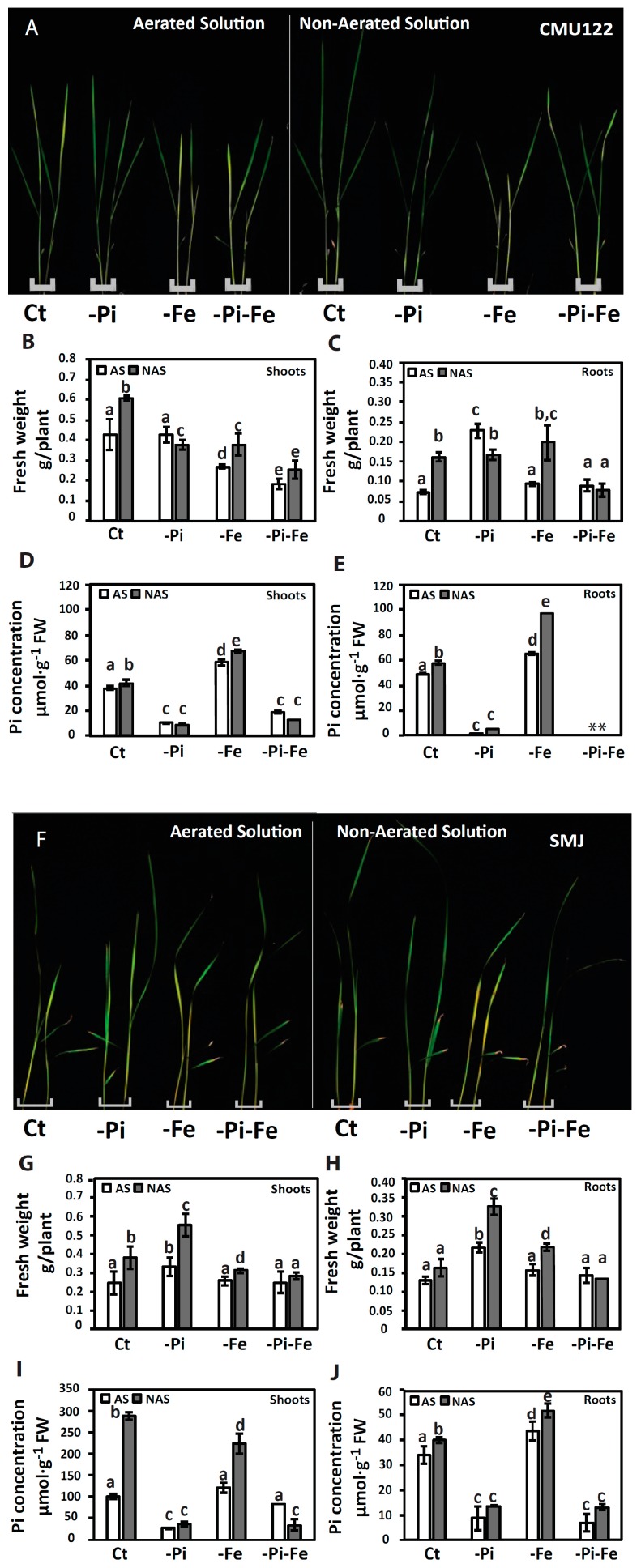
Responses of upland rice (CMU122, Sew Mae Jun (SMJ) varieties) seedlings grown under various O_2_ and nutrient deficiency conditions. Ten-day-old seedlings were transferred to one of the combinatory treatments consisting of O_2_ (aerated solution (AS) and non-aerated solution (NAS)) nutrient treatments (full strength nutrient solution (Ct), phosphate deficiency (−Pi), iron deficiency (−Fe) and Pi and Fe deficiency (−Pi-Fe)). (**A**,**F**) Phenotypes of seedlings after 10 days of treatment; (**B**,**G**) shoots and (**C**,**H**) roots biomass of seedlings after 10 days of treatment; (**D**,**I**) shoots and (**E**,**J**) roots Pi concentration of seedlings after 10 days of treatment. The data are given as mean ± s.e. (*n* = 3). Letters a, b, c, d, e on histograms indicate statistical significance at *p* < 0.05. FW, fresh weight.

After 10 days of treatment under full nutrient conditions, all genotypes displayed significantly higher shoot biomass in the non-aerated conditions compared to those grown under aerated conditions (Figure 1B,G and Figure 2B,G). In these same conditions, root biomass tends to increase in upland rice cultivars (Figure 2C,H) compared to lowland rice cultivars where no significant changes are observed (Figure 1C,H). When grown in -Pi and non-aerated conditions, while CMU122 and SRP1 showed generally rather stable biomass (Figure 1G and Figure 2B), Nipponbare and SMJ plants showed a sharp increase in shoot biomass compared to plants grown in -Pi and aerated conditions (Figure 1B and Figure 2G). These results indicate that Pi and O_2_ signals interact to control shoot growth, and can occur in both lowland and upland rice.

In the presence of Pi and Fe (Ct), the Pi concentrations in shoots and roots increase in both lowland and upland rice grown in non-aerated solution compared to the aerated condition (Figure 1D,E,I,J and Figure 2D,E,I,J, and Table S1). Our analysis shows also that Pi concentration increases under Fe deficiency, depending on the availability of O_2_, regardless the genotype tested (Figure 1D,E,I,J and Figure 2D,E,I,J, and Table S1).

In plants, Fe deficiency manifests itself by leaf chlorosis and affects shoot biomass, which is in agreement to what is observed in all varieties when compared to full nutrient media, in both aerated and non-aerated conditions (Figure 1A,F and Figure 2A,F) [14]. Our data revealed that upland rice varieties (CMU122 and SMJ) are more tolerant to Fe deficiency when compared to lowland rice varieties (Nipponbare and SRP1) regardless of the presence or absence of O_2_ as illustrated by the higher root and shoot biomass observed (Figure 1B,C,G,H and Figure 2B,C,G,H). In Nipponbare and SRP1, shoots and roots biomass showed a sharp and significant decrease in response to Fe deficiency in both aerobic and anaerobic conditions (Figure 1B,C,G,H). However, in CMU122 and SMJ, Fe deficiency was associated with mild changes in biomass compared to control conditions (Figure 2B,C,G,H). It is noteworthy that CMU122 showed a more severe decrease of shoots biomass in non-aerated to aerated conditions compared to SMJ (Figure 2B,G). This result indicates that the Fe deficiency response mechanisms might be inhibited by O_2_ availability in the growth solution (Figure 2B,C,G,H).

Upland and lowland rice cope often with a simultaneous fluctuation in P and Fe availabilities in their environment. Interaction between nutrients homeostasis was demonstrated to influence plant growth [13,14,15,16]. More particularly, it has recently been demonstrated that combination of Pi and Fe deprivation alleviated the negative effect associated with Fe deficiency alone in rice [14]. Here, we confirm the fact that Pi deficiency can alleviate the severe effect of Fe deficiency in Nipponbare and SRP1, in an O_2_-dependant manner (Figure 1A,B,F,G). Indeed, only under non-aerated conditions, the Nipponbare and SRP1 plants grown on −Pi-Fe conditions showed higher shoots and roots biomass in comparison to the same genotypes grown on −Fe alone (Figure 1B,G). Conversely, regardless the availability of O_2_, the shoots and roots biomass of CMU122 and SMJ plants grown in −Pi-Fe condition was not improved compared to −Fe condition (Figure 2B,G). These results indicate that Pi, Fe and O_2_ signals interact to regulate shoots and roots biomass in Nipponbare and SRP1 genotypes. However, in CMU122 and SMJ genotypes, the O_2_ level plays a minor role in Pi–Fe interaction to regulate plant growth. Our results thus reveal a contrasting behavior between the two groups of rice varieties (upland and lowland rice), with ion homeostasis interaction and its effect on roots and shoots biomass.

## 3. Discussion

The knowledge of the existence of interactions between mineral elements has been present for more than 50 years and has been mainly studied at the physiological level. Fortunately, the area is being revisited due to the technological advances in genetic tools and molecular biology. In addition, interactions between mineral nutrients and soil oxygen availability in the soil solution and nutrient availability (P and Fe) in a genotype are starting to emerge. Recent study demonstrated that plant root hydraulics is regulated by a potassium-dependent O_2_ sensing pathway in the model plant *Arabidopsis thaliana* [17]. A molecular pathway involving Hydraulic Conductivity of Root 1 and the transcription factor RAP2 was proposed as a relevant target for improving the resilience of plants to flooding. Genetic and molecular studies would lead to the identification of the precise molecular pathways involved in this mineral nutrients and O_2_ signal crosstalk in rice, and turn this fundamental knowledge about the phenotypic relationships between genotype and environment into applications such as the improvement of Pi and Fe nutrition in rice. Beyond this study, it is worth noting that, in addition to Fe, Pi can interact with other elements such as Zn [13], and should thus be taken into account in future studies [18].

## 4. Materials and Methods

### 4.1. Plant Growth Conditions

This study used four contrasting ecotypes of rice (*Oryza sativa* L.) divided into lowland rice (Nipponbare, SPR1) and upland rice (CMU122, SMJ). The experiments were conducted in a controlled-environment chamber (light/dark cycle of 14/10 h, 200 µmol photons·m^−2^·s^−1^), temperature of 28/25 °C and relative humidity (RH) of 80%). The rice plants were grown in a nutrient solution. Seeds were dehulled and soaked in deionized water overnight in darkness. Then, seedlings were exposed to light for two days and transferred to ¼ of the full strength nutrient solution without air bubbling for eight days. The seedlings were transplanted to 2 L tanks containing nutrient solution with and without air bubbling to simulate aerated (AS) and non-aerated (NAS) conditions. O_2_ concentration in the nutrient solution was measured after having transplanted the seedlings for five days. The percentage of O_2_ was 19.2% ± 0.2% in aerated conditions and 11.6% ± 0.7% in non-aerated conditions, and these two conditions were statistically different at *p* < 0.05 (%O_2_ data were the means of six replicates ± s.e.). These conditions were additionally combined with treatments of nutrient deficiency consisting of phosphate (−Pi), iron (−Fe) and both of Pi and Fe (−Pi-Fe). Full strength nutrient solution was used for control (Ct). The composition of nutrient solution at full concentration was: NH_4_NO_3_, 1.43 mM; MgSO_4_, 1.64 mM; CaCl_2_, 0.75 mM; K_2_SO_4_, 0.51 mM; NaH_2_PO_4_, 0.33 mM; H_3_BO_3_, 20 µM; MnCl_2_, 10 µM; Fe-NaEDTA, 40 µM; ZnSO_4_, 2.5 µM; CuSO_4_, 0.16 µM; and (NH_4_)_6_Mo_7_O_24_, 0.08 µM (modified from Saenchai et al. [14] and Yoshida et al. [19]). In the nutrient solution, NaH_2_PO_4_ as Pi and Fe-NaEDTA as Fe was omitted by single or double reagents as treatment of −Pi, −Fe and −P-Fe. For all of the treatments, the solution contained 2.5 mM MES buffer, was renewed every five days, and pH was adjusted to 6.5 using citric acid. The fresh weight of shoots was measured after 10 days of treatment. Phosphate (Pi) concentration was determined as described by Saenchai et al. [14].

### 4.2. Statistical Analysis

ANOVAs on the data were performed using Statistix 8 (analytical software, SXW, Tallahassee, FL, USA). For all of the *t*-test analyses, the difference is considered statistically significant with a probability of *p* < 0.05.

## 5. Conclusions

In our study using four rice cultivars, we demonstrated the existence of tripartite interactions between Pi, Fe and O_2_ to control growth, which vary between upland and lowland rice cultivars. As a consequence, these types of studies of multiple element interaction would greatly benefit from performing whole ionome analysis in these plants in order to better comprehend the global picture and potentially identify other interactions. In addition, in order to better understand the mechanisms used by rice cultivars for their local adaptation, global gene expression analysis, such as RNA-seq, could help. Finally, understanding the molecular basis of these nutrient signals’ crosstalk is seen as an essential step towards developing novel strategies to create rice varieties with improved capacity to adapt to its environment and increase yield.

## Supplementary Materials

Supplementary materials can be found at www.mdpi.com/1422-0067/18/3/607/s1.

## References

[1] Rehman H., Aziz T., Farooq M., Wakeel A., Rengel Z. (2012). Zinc nutrition in rice production systems: A review. Plant Soil.

[2] Sun F., Kolvenbach B.A., Nastold P., Jiang B., Ji R., Corvini P.F.X. (2014). Degradation and metabolism of Tetrabromobisphenol A (TBBPA) in submerged soil and soil-plant systems. Environ. Sci. Technol..

[3] Voesenek L.A.C.J., Bailey-Serres J. (2015). Flood adaptive traits and processes: An overview. New Phytol..

[4] Serraj R., McNally K.L., Slamet-Loedin I., Kohli A., Haefele S.M., Atlin G., Kumar A. (2011). Drought resistance improvement in rice: An integrated genetic and resource management strategy. Plant Prod. Sci..

[5] Fukao T., Xiong L. (2013). Genetic mechanisms conferring adaptation to submergence and drought in rice: Simple or complex?. Curr. Opin. Plant Biol..

[6] Mongon J., Konnerup D., Colmer T.D., Rerkasem B. (2014). Responses of rice to Fe^2+^ in aerated and stagnant conditions: Growth, root porosity and radial O_2_ loss barrier. Funct. Plant Biol..

[7] Atwell B.J., Greenway H., Colmer T.D. (2015). Efficient use of energy in anoxia-tolerant plants with focus on germinating rice seedlings. New Phytol..

[8] Colmer T.D., Cox M.C.H., Voesenek L.A.C.J. (2006). Root aeration in rice (*Oryza sativa*): Evaluation of O_2_, carbon dioxide, and ethylene as possible regulators of root acclimatizations. New Phytol..

[9] Voesenek L.A.C.J., Bailey-Serres J. (2013). Flooding tolerance: O_2_ sensing and survival strategies. Curr. Opin. Plant Biol..

[10] Colmer T.D. (2003). Aerenchyma and an inducible barrier to radial O_2_ loss facilitate root aeration in upland, paddy and deep-water rice (*Oryza sativa* L.). Ann. Bot..

[11] Secco D., Bouain N., Rouached A., Prom-u-thai C., Hanin M., Pandey A.K., Rouached H. (2017). Phosphate, phytate and phytases in plants: From fundamental knowledge gained in *Arabidopsis* to potential biotechnological applications in wheat. Crit. Rev. Biotechnol..

[12] Heuer S., Gaxiola R., Schilling R., Herrera-Estrella L., Lopez-Arredondo D., Wissuwa M., Delhaize E., Rouached H. (2016). Improving phosphorus use efficiency—A complex trait with emerging opportunities. Plant J..

[13] Briat J., Rouached H., Tissot N., Gaymard F., Dubos C. (2015). Integration of P, S, Fe and Zn nutrition signals in *Arabidopsis thaliana*: Potential involvement of PHOSPHATE STARVATION RESPONSE1 (PHR1). Front. Plant Sci..

[14] Saenchai C., Bouain N., Kisko M., Prom-u-thai C., Doumas P., Rouached H. (2016). The involvement of OsPHO1;1 in the regulation of iron transport through integration of phosphate and zinc deficiency signaling. Front. Plant Sci..

[15] Zheng L., Huang F., Narsai R., Wu J., Giraud E., He F., Cheng L., Wang F., Wu P., Whelan J. (2009). Physiological and transcriptome analysis of iron and phosphorus interaction in rice seedlings. Plant Physiol..

[16] Bouain N., Kisko M., Rouached A., Dauzat M., Lacombe B., Belgaroui N., Ghnaya T., Davidian J.C., Berthomieu P., Abdelly C. (2014). Phosphate/zinc interaction analysis in two lettuce varieties reveals contrasting effects on biomass, photosynthesis, and dynamics of Pi transport. BioMed Res. Int..

[17] Shahzad Z., Canut M., Tournaire-Roux C., Martinière A., Boursiac Y., Loudet O., Maurel C. (2016). A potassium-dependent oxygen sensing pathway regulates plant root hydraulics. Cell.

[18] Rouached H., Secco D., Arpat B.A. (2010). Regulation of ion homeostasis in plants: Current approaches and future challenges. Plant Signal. Behav..

[19] Yoshida S., Foorno D.A., Cock J.H., Gomez K.A. (1976). Laboratory Manual for Physiological Studies of Rice.

