# Supplementary material for: Phosphorus and Iron Deficiencies Influences Rice Shoot Growth in an Oxygen Dependent Manner: Insight from Upland and Lowland Rice
Source: Int J Mol Sci. 2017 Mar 10;18(3):607. doi: 10.3390/ijms18030607 (PMC5372623; doi:10.3390/ijms18030607)
Supplement: Supplementary file 1 [file ijms-18-00607-s001.pdf]

Pi (µmol.gFW-1)

Shoots	Treatments	Nipponbare				SPR1			
		AS		NAS		AS		NAS	
	Ct	13,369	±0,79	17,15	±1,69	39,5	±2,98	71,0	±3,18
	-Pi	4,011	±0,30	6,90	±0,98	9,4	±0,86	10,1	±0,96
	-Fe	33,722	±1,75	38,91	±1,86	66,9	±1,90	86,7	±3,05
	-Pi-Fe	6,747	±1,76	4,95	±0,90	27,5	±0,21	8,7	±0,70

Roots	Treatments	Nipponbare				SPR1			
		AS		NAS		AS		NAS	
	Ct	9,043	±0,77	14,13	±0,88	15,9	±0,75	36,3	±0,38
	-Pi	0,198	±0,09	3,42	±0,59	2,1	±0,11	2,6	±0,20
	-Fe	8,930	±0,26	15,91	±0,87	43,9	±0,70	49,5	±3,35
	-Pi-Fe	0,120	±0,04	1,92	±0,21	1,4	±0,16	2,3	±0,46

Shoots	Treatments	CMU122				SMJ			
		AS		NAS		AS		NAS	
	Ct	37,76	±1,24	42,08	±2,3	81,26	±5,31	231,11	±6,88
	-Pi	10,13	±0,9	8,96	±0,69	21,24	±0,94	29,38	±4,68
	-Fe	59,74	±2,63	67,07	±0,91	96,64	±9,19	179,97	±19,09
	-Pi-Fe	18,94	±1,04	12,72	±0,84	67,03	±0,23	27,68	±10,55

Roots	Treatments	CMU122				SMJ			
		AS		NAS		AS		NAS	
	Ct	49,20	±0,74	56,96	±1,68	33,72	±3,48	39,85	±1,2
	-Pi	2,53	±0,14	5,35	±0,32	8,57	±4,72	13,53	±0,21
	-Fe	63,49	±0,29	97,65	±1,56	41,97	±4,3	51,67	±2,8
	-Pi-Fe	0,44	±0,12	0,50	±0,46	6,89	±3,47	13,03	±1,07
